# Maternal karyogene and cytoplasmic genotype affect the induction efficiency of doubled haploid inducer in *Brassica napus*

**DOI:** 10.1186/s12870-021-02981-z

**Published:** 2021-05-03

**Authors:** Wei Zhang, Yongting Ma, Zhendong Zhu, Liangjun Huang, Asif Ali, Xuan Luo, Ying Zhou, Yun Li, Peizhou Xu, Jin Yang, Zhuang Li, Haoran Shi, Jisheng Wang, Wanzhuo Gong, Qiong Zou, Lanrong Tao, Zeming Kang, Rong Tang, Zhangjie Zhao, Zhi Li, Shixing Guo, Shaohong Fu

**Affiliations:** 1 Chengdu Academy of Agricultural and Forestry Sciences, Chengdu, 611130 China; 2 Chengdu Research Branch, National Rapeseed Genetic Improvement Center, Chengdu, 611130 China; 3 Agricultural College, Sichuan Agricultural University, Chengdu, 611130 China; 4 Rice Research Institute, Sichuan Agricultural University, Chengdu, 611130 China; 5 Maize Research Institute, Sichuan Agricultural University, Chengdu, 611130 China

**Keywords:** Double haploid inducer, Paternal infiltration, Chromosome elimination, Polyploid, *Brassica napus*

## Abstract

**Background:**

Artificial synthesis of octoploid rapeseed double haploid (DH) induction lines Y3380 and Y3560 was made possible by interspecific hybridization and genome doubling techniques. Production of pure lines by DH induction provides a new way to achieve homozygosity earlier in *B.napus*. Previously, the mechanism of induction, and whether the induction has obvious maternal genotypic differences or not, are not known so far.

**Results:**

In this study, different karyogene and cytoplasmic genotype of *B.napus* were pollinated with the previously reported DH inducers e.g. Y3380 and Y3560. Our study presents a fine comparison of different cytoplasmic genotypes hybridization to unravel the mechanism of DH induction. Ploidy identification, fertility and SSR marker analysis of induced F_1_ generation, revealed that ploidy and phenotype of the induced F_1_ plants were consistent with that type of maternal, rather than paternal parent. The SNP chip analysis revealed that induction efficiency of DH inducers were affected by the karyogene when the maternal cytoplasmic genotypes were the same. However, DH induction efficiency was also affected by cytoplasmic genotype when the karyogenes were same, and the offspring of the *ogura* cytoplasm showed high frequency inducer gene hybridization or low-frequency infiltration.

**Conclusion:**

The induction effect is influenced by the interaction between maternal karyogene and cytoplasmic genotype, and the results from the partial hybridization of progeny chromosomes indicate that the induction process may be attributed to the selective elimination of paternal chromosome. This study provides a basis for exploring the mechanism of DH inducer in *B.napus*, and provides new insights for utilization of inducers in molecular breeding.

**Supplementary Information:**

The online version contains supplementary material available at 10.1186/s12870-021-02981-z.

## Background

*Brassica napus* (AACC, 2n = 4 × =38) is an allotetraploid plant derived from *Brassica rape* (AA, 2n = 20) and *Brassica oleracea* (CC, 2n = 18) through interspecific cross and natural doubling of chromosomes that happened about 7500 years ago [1, 2]. Breeding of *B.napus* varieties has appreciated the utilization of heterosis that mainly includes different technical methods e.g. *polima* cytoplasmic sterility (*pol* CMS) and *ogura* cytoplasmic sterility (*ogu* CMS). Before the advent of successful application of microspore culture in *B.napus*, the pure lines were used to obtained by means of multi-generational selfing [3–5], but breeding cycle was long. Although isolated microspore culture can speed up the breeding cycle, it is limited by many factors such as genotype and environmental temperature [6–8]. Rapeseed scientists are keen to find that is there a simpler and more efficient technique than isolated microspore culture that can quickly and efficiently obtain pure lines of *B.napus*? In recent years, in-vivo haploid induction line was derived from the maize *Stock6* [9] and the haploid induction line mediated by the *Arabidopsis CENH3* gene have been successfully used in maize [10], barley [11], and rice [12]. Maize haploid induction gene *ZmPLA1* [13] and *Arabidopsis* gene *CENH3* [14–16] have been applied to wheat, and haploid induction have been achieved. In addition, the use of barley bulb method and distant pollination of corn pollens in wheat have also achieved haploid induction, in which induction rate was about 20–45% [14]. It is easier to get pure lines induced by crossing with haploids than isolated microspore culture. A recent study reported, in which an allotetraploid *B.napus* was crossed with an allo-octaploid rape (AAAACCCC, 2n = 8x ≈ 76) [17] and two allo-octaploid rapes had induced the function of the maternal parent to produce double haploids (DH) and named as the DH induction lines in *B.napus*: Y3560 and Y3380 [18]. SSR molecular markers, plant ploidy, and morphological identification revealed that a higher proportion of plants in the F_1_ generation were similar to the maternal parent and induction efficiency ranges from 34.09% ~ 98.66%. What accounts for such a huge difference in induction efficiency? And it was observed that there were different induction effects according to maternal cytoplasmic genotypes of *B.napus*. Whether the induction efficiency was related to the cytoplasmic genotype of the maternal parent? Therefore, in this study we used DH inducer lines as paternal parent to pollinate three types of *pol*, *ogu* and *nap* cytoplasmic maternal parents, and ploidy, phenotype and genotypes of the induced offspring were observed. SNP analysis was performed to evaluate the relationship of maternal parent cytoplasmic effect. This study lays the foundation for the application of the DH lines and contributes to the understanding of maternal karyotype and cytoplasmic genotype effects.

## Results

### Ploidy analysis

**Fig. 1:**
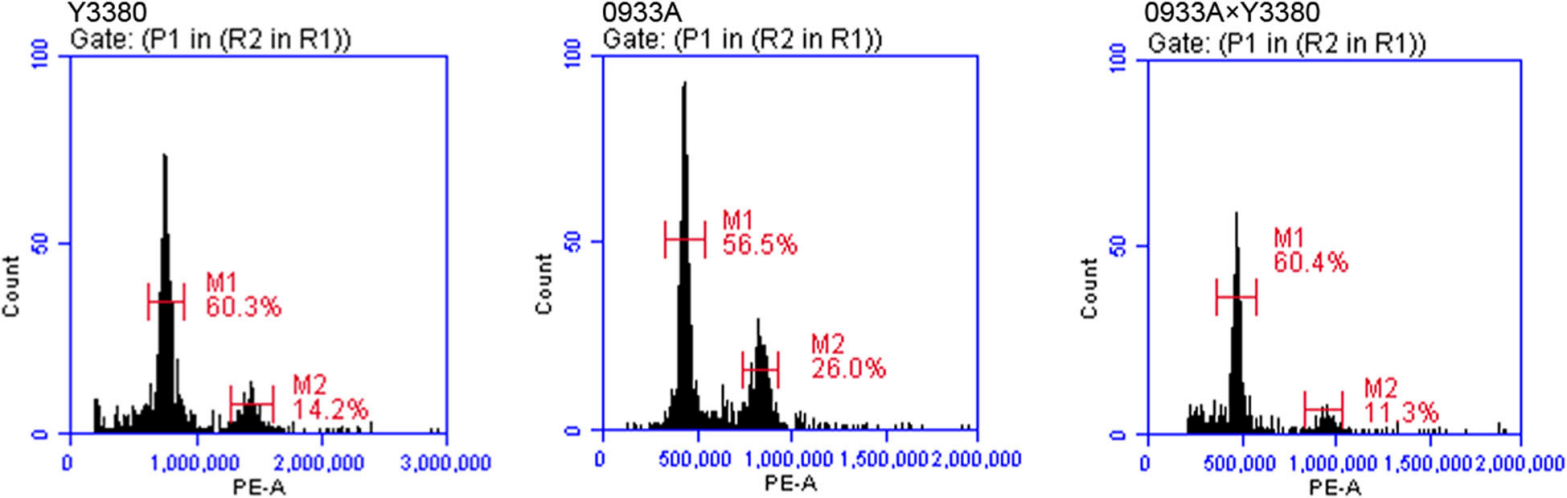
Flow cytometry diagram of induced line Y3380, maternal parent 0933A and their induced offspring (0933A × Y3380)

The offspring of a hybrid between high-ploidy and low-ploidy are easily prone to an intermediate ploidy [19]. In order to understand whether there were differences in ploidy level before and after induction, we selected different *B.napus* induced F_1_ and tetraploid *B.napus* ZS11 that was used as control. The detection results of ploidy are given as follows (Fig. 1, Additional file 1, Additional file 2). The fluorescence intensity of F_1_ generation obtained by pollination of Y3560 and four maternal parents was about 409.5 ~ 510.5D thousand lines. The fluorescence intensity of F_1_ generation of Y3380 pollinated with five maternal parents was about 398.9 ~ 521.1D thousand lines, and the detection results were almost the same as peak value of 423.1 ~ 487.7D thousand lines in control hybrid offspring. We also tested the ploidy of pollinated maternal parents and DH inducer. The fluorescence intensity of the maternal parent plants ranged from 406.9 ~ 502.1D thousand lines all of them were tetraploid. While, the fluorescence intensity of inducer was about 753.2 ~ 852.5D thousand lines, which was two times higher than that of ZS11 (control), hence inducer were octoploid. In this study we selected octoploid rapeseed DH lines as the paternal parent and crossed with tetraploid *B.napus*. It was found that ploidy of offspring was consistent that of maternal ploidy (tetraploid). In order to find the preliminary explanation of octoploid and tetraploid cross produced a tetraploid encouraged us to study how an octoploid paternal parent may have played an induced or partial chromosomal hybrid effect?

### Plant morphology and fertility investigation

**Fig. 2:**
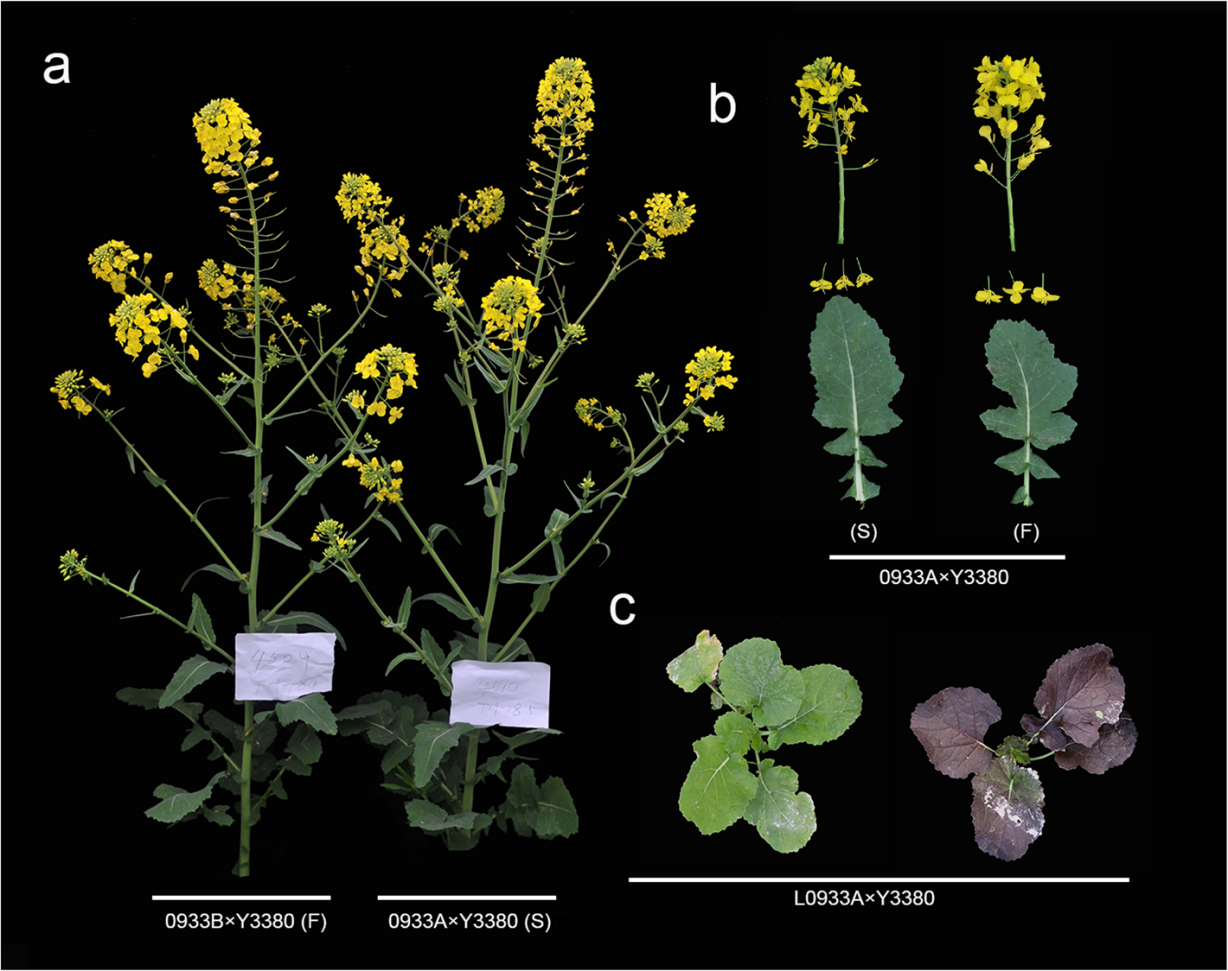
Comparison of phenotype in florescence flower and leaf of 0933. **a**: Phenotype of maternal parent 0933B × Y3380 and 0933A × Y3380 (F: fertility, S: sterility). **b**: Phenotype of inflorescence and flower of 0933A × Y3380 (F: fertility, S: sterility). **c**: Phenotype of leaf of induction line L0933A progeny with leaf contrasts in purple color

In the previous study, it was found that a high percentage of the induced F_1_ generation plants were similar to the maternal parent, but the fertility of some of them were changed. So, the morphology and fertility of the induced F_1_ generation plants were observed to investigate the effect of DH induction. Fertility and plant morphology investigations (Fig. 2, Additional file 3) of F_1_ showed that most of plants were sterile, while only a few were fertile. Sterile lines were pollinated by the DH inducer lines (paternal parent), while those of the fertile plants have shown no obvious difference from the maternal parents except for the flower morphology. Fertility identification of paternal parent Y3560, Y3380 and induced F_1_ were given in follows Additional file 3. The homozygous and stable *pol* CMS of 0068A, 0933A and D717A were used as the maternal parent and crossed with Y3560, and as a result six, two, and one fertile plant were found in their offspring, respectively. Only one fertile plant was found in F_1_, when Y3380 was used as the paternal parent and homozygous *pol* CMS 0933A was used as maternal parent. There were no fertile plants found in F_1_, when Y3380 was crossed with L0068A and L0933A as maternal parent. At same time, the hybrid progeny was also developed by crossing ZS11 as paternal parent with different maternal parents, and all resulting progeny produced were semi-sterile or sterile. Results of fertility identification revealed that among the induced offspring from paternal parents Y3560 and Y3380, the fertile plants appeared in the offspring of *pol* CMS, when it was used as maternal parent with a probability of 2.22 to 30.00%. Among them, the probability of 0068A × Y3560 was highest (30.00%) while lowest (2.22%) was found in D717A × Y3560. The induction of fertile plants in *pol* CMS maternal parent may be attributed to the hybridization compatibility of Y3380 and Y3560 with *pol* cytoplasmic restoration genes. According to the probability of fertile plants, the induction rate of Y3380 and Y3560 was estimated to vary from 70 to 100% suggesting that the induction effect of the induced lines may be influenced by the maternal karyotype and cytoplasm type.

### Amplification of of SSR molecular markers and SNP chip analysis of F_1_induction line

**Fig. 3:**
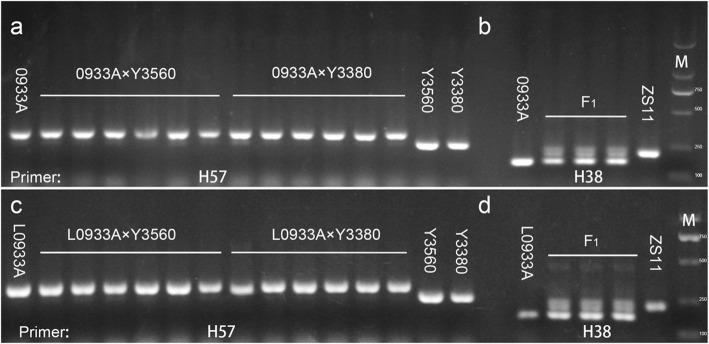
Amplification results of SSR primers of some parents and F_1_ individual plants. **a**: Amplification of SSR specific primer H57 in the paternal inducible line (as paternal parent), 0933A (as maternal parent) and induced progeny, 0933A × Y3560 progeny, from left to right are M31–1, M31–2, M31–5, M31–7, M31–8, M31–12; 0933A × Y3380 progeny, from left to right are M30–1, M30–2, M30–3, M30–6, M30–10, M30–11. **b**: Amplification of specific SSR primer H38 in parents and hybrid progeny (0933A × ZS11). **c**: Amplification of SSR specific primer H57 in the paternal inducible line (as paternal parent), L0933A (as maternal parent) and induced progeny, L0933A × Y3560 progeny,from left to right are M35–1, M35–7, M35–9, M35–10, M35–12, M35–14; L0933A × Y3380 progeny, from left to right are M34–1, M34–2, M34–3, M34–5, M34–6, M34–9. d: Amplification of specific SSR primer H38 in parents and hybrid progeny (L0933A × ZS11). The samples derive from the same experiment and the full-length original gel is included in Additional file 5

**Fig. 4:**
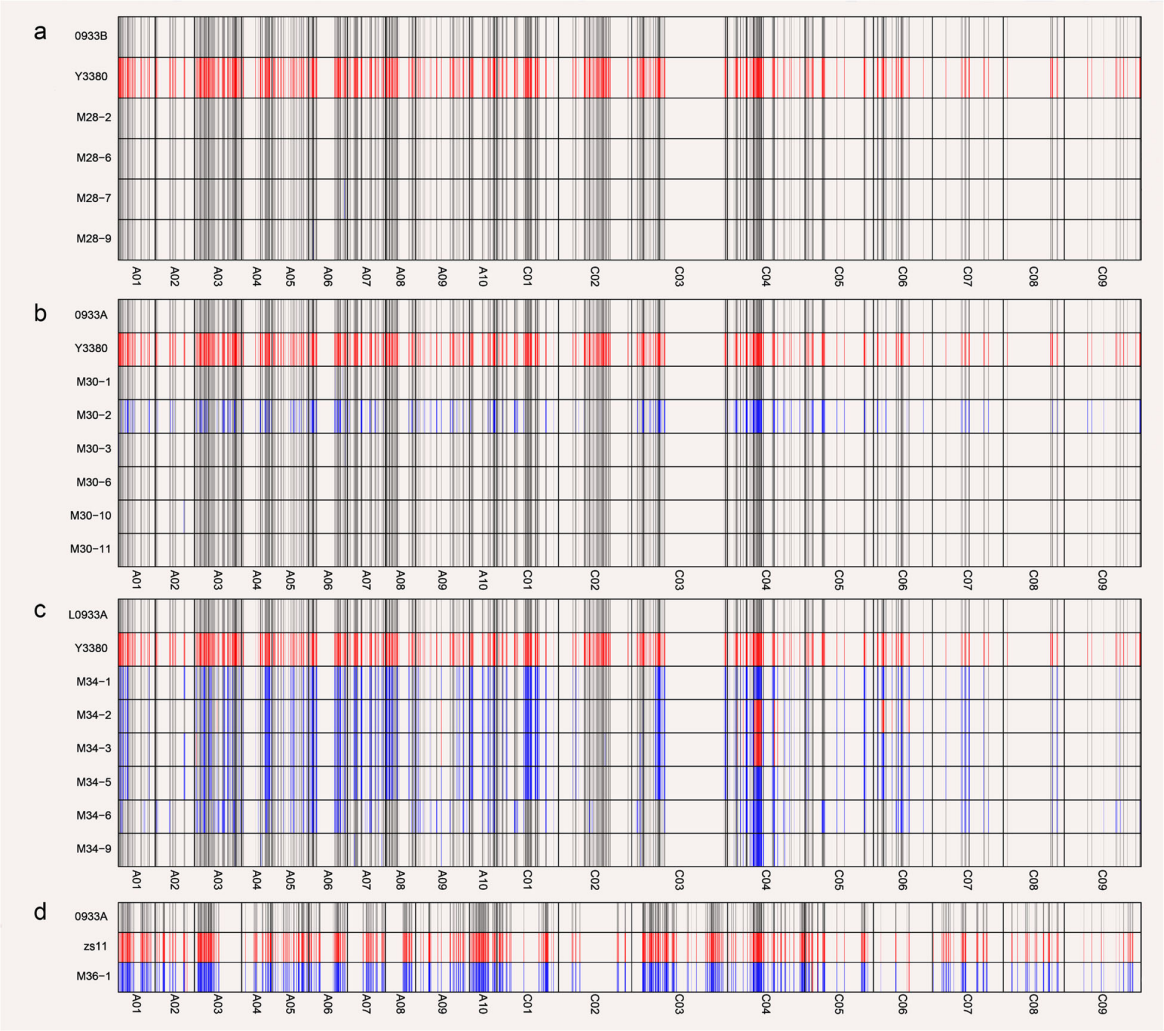
Genotyping diagram of induced line before and after induction. **a**: 0933B × Y3380. **b**: 0933A × Y3380. **c**: L0933A × Y3380. d: 0933A × ZS11. Schematic diagram of the genotyping of parents and progeny, the numbered band M is the progeny plant

Since the morphology and ploidy of the F_1_ generation plants after induction were consistent with that of maternal parents, but it was not clear whether there were any genotypic difference? Firstly, 600 pairs of SSR primers were used to find the polymorphism between parental and maternal parents and their progeny (Fig. 3, Additional file 4, Additional file 5). When Y3560 was used the parental parent, three pairs of specific primers were amplified with 0068A; four pairs of specific primers were amplified with 0933A and L0933A. Those specific primers were also amplified in their offspring in order to reveal the genotypic polymorphism. Amplification of SSR markers revealed that induced F_1_ generation plants were consistent with maternal band type, without paternal or heterozygous band type. At the same time, combined with the field fertility survey, the M1–1, M1–8 and M1–9, the progeny of 0068A × Y3560 were tentatively judged as the induced plants. While for Y3380, three pairs of specific primers were amplified with L0068A, five pairs of specific primers were amplified with 0933A and L0933A, and amplification results showed that in addition to L0068A× Y3380 offspring M4–5, M4–9 revealed heterozygous band type, while remaining offspring showed maternal band type. When ZS11 was used a parental parent, and 2–3 pairs of specific primers were amplified in each of maternal parent. The amplification of primers revealed that the progeny contained the band type of both parents, hence all were hybrid plants. Since the polymorphic primers were not enough, so SNP chip analysis covering more marker sites in whole of genome should be used to verify whether the progeny of each combination is homozygous or heterozygous induced plant. In view of the fact that ploidy, morphology and SSR molecular marker identification would not produce precise results, 62 plants were selected for 6 K SNP chip analysis (Additional files 6). The results of SNP sites homozygosity rate and maternal genetic similarity are given in Additional file 7, and maternal parents used this study showed a homozygosity rate (97.93% ~ 99.29%). Such a higher homozygosity rate was close to that of ZS11 (99.16%), suggesting that these seven maternal parents were almost homozygous. The homozygosity rate of F_1_ hybrid was 63.73% ~ 68.74%, and genotyping (Fig. 4d, Additional file 9c, Additional file 10e) also indicated that these offspring were F_1_ hybrids produced by ZS11 as the paternal parent. At the same time, the homozygosity rate of some F_1_ plants after induction ranged from 56.39% ~ 78.87%, and genotyping (Fig. 4a-c, Additional file 9a-b, Additional file 10a-d) was used to identify the F_1_ as hybrid or partial chromosome hybrid offspring. The rate of homozygous SNP sites in the remaining single plants ranged from 98.48% ~ 99.33%. Genotyping (Fig. 4a-c, Additional file 9a-b, Additional file 10a-d) confirmed that these F_1_ were homozygous plants. Therefore, the analysis of the homozygosity rate between different individual plants showed that when DH lines were used as paternal parent, and those offspring, who produced a homozygosity rate of about 60%were hybrid offspring, and while thosee witha homozygosity rate > 95% were the induced offspring. Subsequently, we analyzed the genetic distance between the maternal parent and 62 (F_1_) plants, and calculated the genetic similarity rate between the maternal parents and their F_1_ offspring. The genetic similarity rate between hybrid offspring and the maternal parent was 64.64% ~ 68.74%, while that of genetic similarity rate between the induced homozygous individual plant and the maternal parent was as high as 99.33%. Hence, It was confirmed that the induction induced by the DH inducer was not an ordinary cross, and the F_1_ generation produced was the same homozygous offspring as the maternal parent.

### Genotyping revealed paternal chromosome hybridization and infiltration during induction process

**Fig. 5:**
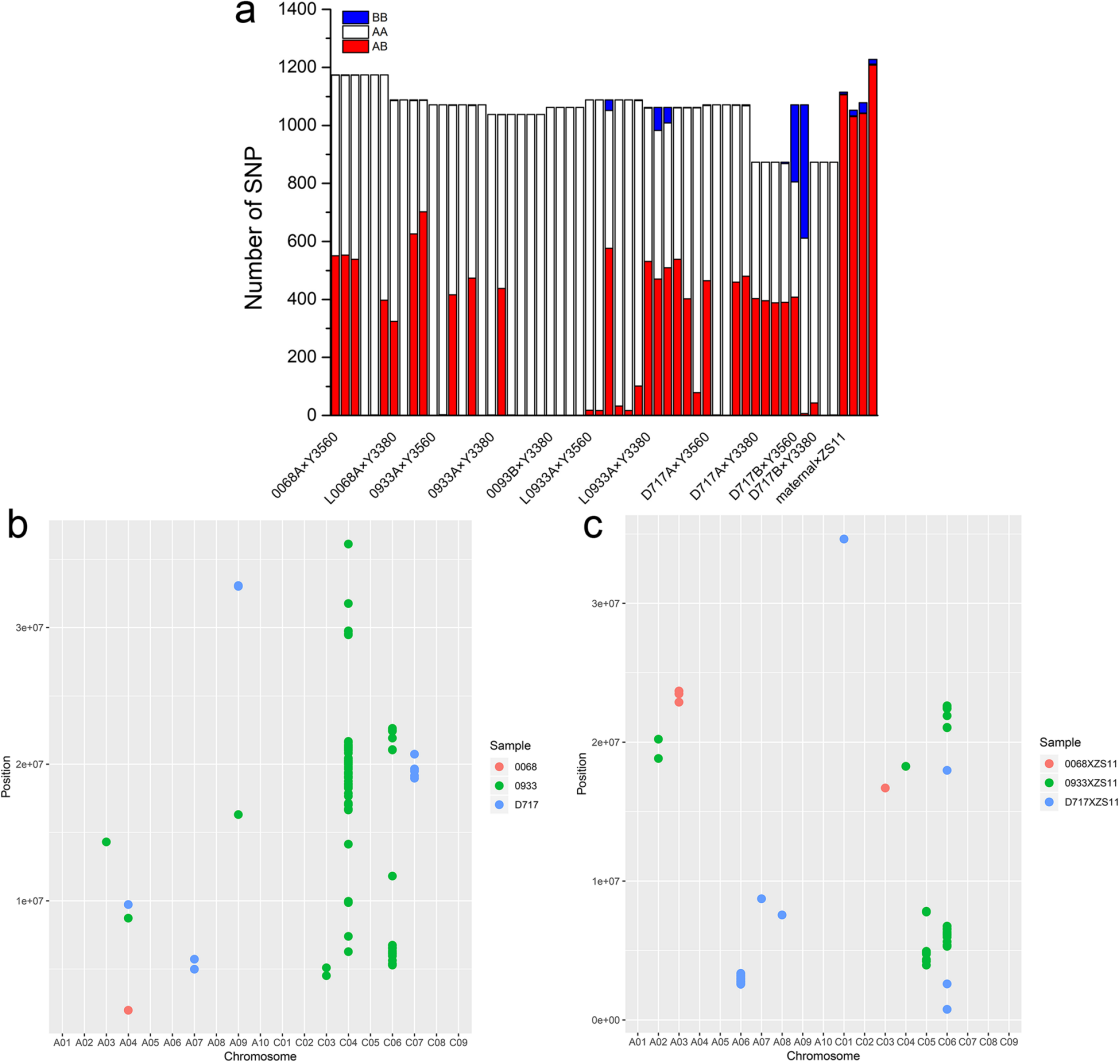
Induction line induction effect and infiltrated SNP quantity typing statistics. **a**: Induced line induction effect SNP quantity typing statistics; AA represents the same SNP site as the maternal parent, BB represents the same SNP site as the paternal parent, and AB represents the heterozygous site. **b**: The distribution of the parental infiltrating SNP loci on each chromosome of the induced progeny after induction, when DH inducer line was used as the paternal parent. The horizontal coordinates are chromosomes and the vertical coordinates are the chromosome positions, where the SNP loci were located, and different colors represent different maternal parents. **c**: The distribution of the parental infiltrating SNP loci on each chromosome of the hybrid progeny after crossing, using ZS11 as the paternal parent. The horizontal coordinates are chromosomes and the vertical coordinates are the chromosome positions where the SNP loci were located, and different colors represent different maternal parents

In order to better understand the paternal chromosome hybridization and infiltration during induction process, homozygous SNP sites were screened and genotyping revealed SNP sites in offspring (Fig. 4, Additional file 9, Additional file 10, Additional file 11). The number of homozygous SNP sites in parental differences ranged from 873 to 1227 (Table 1, Fig. 5a, Additional file 11). Taking the maternal parent 0933 as an example, compared with the hybrid combination 0933A× ZS11 (Fig. 4d) revealed 97.91% heterozygosity, when 0933B (Fig. 4a) and 0933A (Fig. 4b) were the maternal parent, genotype of the induced offspring was consistent with those of the maternal parent. Hybridization of 33.84% ~ 44.26% paternal genes and paternal infiltration rate of 0.09 ~ 0.19% were observed in the hybrid offspring. When L0933A (Fig. 4c) was used as maternal parent, the hybrid offspring revealed 37.87% ~ 53.03% (Table 1) paternal genes heterozygosity and 0.09% ~ 0.18% (Table 1) paternal introgression. These results indicated that the induction efficiency was influenced by cytoplasmic genotype, and the hybridization of *ogu* cytoplasmic genotype was more prone to occur more like maternal parent when the karyogene was same. When the cytoplasmic genotype was *pol* (0068A, 0933A, D717A), the offspring of 0068A (33.82% ~ 47.10%) (Table 1) were more likely to cross with the paternal parent; and when the cytoplasmic type was *nap* (0933B, D717B), the offspring of D717B (24.84% ~ 42.86%) (Table 1) were most likely to infiltrate the paternal gene. While, the cytoplasm genotype was *ogu* (L0068A, L0933A), the L0933A was easier to cross or exchange with the paternal parent, and was more likely to be on the C-genome (Fig. 5b), indicating that when cytoplasm genotype was the same, the induction effect was affected by the maternal parent nuclear genotype. In conclusion, the induction efficiency is influenced by both the maternal karyotype and the cytoplasm genotype, and effect of karyotype > cytoplasmic genotype.

**Table 1:** Statistics of genotyping SNP sites

Sample source	sample name	SNP number	Heterozygous number	Heterozygosity rate(%)	maternal number	maternal infiltration rate(%)	paternal number	paternal infiltration rate(%)
0068A × Y3560	M1–1	1174	551	46.93	622	52.98	1	0.09
	M1–2	1174	553	47.10	619	52.73	2	0.17
	M1–3	1174	539	45.91	634	54.00	1	0.09
	**M1–8**	**1174**	**0**	**0.00**	**1174**	**100.00**	**0**	**0.00**
	**M1–9**	**1174**	**2**	**0.17**	**1172**	**99.83**	**0**	**0.00**
	M1–10	1174	397	33.82	777	66.18	0	0.00
L0068A × Y3380	M4–3	1088	324	29.78	762	70.04	2	0.18
	**M4–4**	**1088**	**0**	**0.00**	**1088**	**100.00**	**0**	**0.00**
	M4–5	1088	626	57.54	460	42.28	2	0.18
	M4–9	1088	702	64.52	385	35.39	1	0.09
0068A × ZS11	*M5–1*	*1115*	*1106*	*99.19*	*3*	*0.27*	*6*	*0.54*
0933A × Y3560	**M31–1**	**1071**	**1**	**0.09**	**1070**	**99.91**	**0**	**0.00**
	**M31–2**	**1071**	**3**	**0.28**	**1068**	**99.72**	**0**	**0.00**
	M31–5	1071	416	38.84	654	61.06	1	0.09
	**M31–7**	**1071**	**1**	**0.09**	**1070**	**99.91**	**0**	**0.00**
	M31–8	1071	474	44.26	595	55.56	2	0.19
	**M31–12**	**1071**	**0**	**0.00**	**1071**	**100.00**	**0**	**0.00**
0933A × Y3380	**M30–1**	**1038**	**2**	**0.19**	**1036**	**99.81**	**0**	**0.00**
	M30–2	1038	438	42.20	599	57.71	1	0.10
	**M30–3**	**1038**	**2**	**0.19**	**1036**	**99.81**	**0**	**0.00**
	**M30–6**	**1038**	**0**	**0.00**	**1038**	**100.00**	**0**	**0.00**
	**M30–10**	**1038**	**1**	**0.10**	**1037**	**99.90**	**0**	**0.00**
	**M30–11**	**1038**	**0**	**0.00**	**1038**	**100.00**	**0**	**0.00**
0093B × Y3380	**M28–2**	**1062**	**0**	**0.00**	**1062**	**100.00**	**0**	**0.00**
	**M28–6**	**1062**	**1**	**0.09**	**1061**	**99.91**	**0**	**0.00**
	**M28–7**	**1062**	**1**	**0.09**	**1061**	**99.91**	**0**	**0.00**
	**M28–9**	**1062**	**1**	**0.09**	**1061**	**99.91**	**0**	**0.00**
L0933A × Y3560	**M35–1**	**1088**	**18**	**1.65**	**1070**	**98.35**	**0**	**0.00**
	**M35–7**	**1088**	**17**	**1.56**	**1071**	**98.44**	**0**	**0.00**
	M35–9	1088	577	53.03	475	43.66	36	3.31
	**M35–10**	**1088**	**33**	**3.03**	**1055**	**96.97**	**0**	**0.00**
	**M35–12**	**1088**	**17**	**1.56**	**1071**	**98.44**	**0**	**0.00**
	M35–14	1088	102	9.38	984	90.44	2	0.18
L0933A × Y3380	M34–1	1062	531	50.00	529	49.81	2	0.19
	M34–2	1062	470	44.26	513	48.31	79	7.44
	M34–3	1062	509	47.93	500	47.08	53	4.99
	M34–5	1062	539	50.75	522	49.15	1	0.09
	M34–6	1062	402	37.85	659	62.05	1	0.09
	**M34–9**	**1062**	**79**	**7.44**	**982**	**92.47**	**1**	**0.09**
0933A × ZS11	*M36–1*	*1053*	*1031*	*97.91*	*2*	*0.19*	*20*	*1.90*
L0933A × ZS11	*M36–1*	*1079*	*1041*	*96.48*	*2*	*0.19*	*36*	*3.34*
D717A × Y3560	M39–2	1071	465	43.42	604	56.40	2	0.19
	**M39–4**	**1071**	**2**	**0.19**	**1069**	**99.81**	**0**	**0.00**
	**M39–6**	**1071**	**1**	**0.09**	**1070**	**99.91**	**0**	**0.00**
	M39–8	1071	460	42.95	610	56.96	1	0.09
	M39–13	1071	480	44.82	588	54.90	3	0.28
D717A × Y3380	M38–1	873	403	46.16	470	53.84	0	0.00
	M38–2	873	396	45.36	477	54.64	0	0.00
	M38–5	873	388	44.44	485	55.56	0	0.00
	M38–9	873	390	44.67	478	54.75	5	0.57
D717B × Y3560	M10–1	1071	408	38.10	397	37.07	266	24.84
	**M10–2**	**1071**	**7**	**0.65**	**605**	**56.49**	**459**	**42.86**
D717B × Y3380	M9–1	873	43	4.93	830	95.07	0	0.00
	**M9–2**	**873**	**1**	**0.11**	**872**	**99.89**	**0**	**0.00**
	**M9–3**	**873**	**2**	**0.23**	**871**	**99.77**	**0**	**0.00**
D717A × ZS11	*M42–1*	*1227*	*1208*	*98.45*	*3*	*0.24*	*16*	*1.30*

Bold text represents induced offspring. Italic text is the hybrid offspring of ZS11 as the paternal parent

### Analysis of the interaction effect between the inducer line and the maternal karyogene

Since maternal karyogene and cytoplasm genotype jointly affects the induction efficiency, it is not clear whether the inducer hybridization and infiltration of the sites is random? Based on the comparison of SNP in genotyping (Table 1, Fig. 5a), it was found that the SNP sites of hybrid offspring (when ZS11 was used as paternal parent) were heterozygous with the exception of individual sites infiltrated by the paternal parents, and the heterozygosity rate were 96.48% ~ 99.19% (Table 1, Fig. 5a), and the paternal infiltration rate was 0.54% ~ 3.34% (Table 1, Fig. 5a). While, using the inducer as the paternal parent, although hybrid progeny will be produced, the heterozygous rate of these hybrid progeny is only 29.78% ~ 64.52% (Table 1, Fig. 5a), and most of the sites are the same as that of maternal parent. Subsequent analysis of the chromosomes in which these hybridization sites are located found that when ZS11 was the paternal parent, hybridization mainly occurs on the C03 and C04 chromosomes of the C genome, and the heterozygosity rate is as high as 99.2% ~ 100% (Additional file 12); When the inducer was used as the paternal parent, although similarly, it mainly occurred on the C03 and C04 chromosomes of the C genome, but the heterozygosity rate on C03 and C04 in the hybrid progeny was 28.1% ~ 68.14 and 30.71% ~ 86.2% (Additional file 12), which were significantly lower than the normal hybridization level. Therefore, the cross produced by using the inducer as the paternal parent is not an ordinary cross.

Meanwhile, the chromosome where the paternal infiltration sites were located were analyzed (Fig. 5b-c, Additional file 13). 0068 and 0933 have similar genetic background (from part of same parent) and used as maternal parent, inducer as paternal parent, the infiltration of the paternal parent gene was more likely to be on the C04 and C06 chromosomes of the C genome (Fig. 5b); while, when ZS11 was the paternal parent, the sites of paternal parent infiltration were mainly on the C05 and C06 chromosomes of the C-genome, and the paternal parent of the inducer is more likely to cause the infiltration (Fig. 5c). However, since the site on chromosome C06 mostly the same site, thus eliminating C06 penetration site on the chromosome (Fig. 5b-c, Additional file 13), and presumed DH lines is more likely to cause parental gene penetration on the C04 chromosome of the C genome. During paternal infiltration caused during normal hybridization is more likely to occur on the C05 chromosome. When the maternal parent was D717, the paternal parent infiltration sites of the inducer was more likely to appear on the C07 chromosome of the C genome, while that of ZS11 was mainly on the A06 chromosome of the A genome and the C06 chromosome of the C genome. In summary, the analysis of the paternal cross and infiltration sites, it is indicated that the DH inducer has an interaction effect with the maternal karyogene, and has the biases on different genomes.

## Discussion

### Induction effect of rape DH inducer lines

When the Maize haploid inducer line induces the maternal plant to produce the haploid, it has obvious maternal genotype influence, the induction efficiency ranges from 2 to 15% [20, 21], there is also about 2% of the paternal gene infiltration phenomenon, and whether the *Bassica napus* DH inducer lines has a similar situation has not been reported. In this study, two DH inducer lines of *B.napus*, Y3380 and Y3560, were used for pollination of maternal parents with different cytoplasmic genotype, and multiple methods were used for identification (Additional file 14). Identification methods were consistent to reflect the effects of induction and hybridization, fertile offspring, heterozygous offspring marker with SSR, and heterozygous offspring detected with SNP, indicating that the detection results were completely reliable. Combining these test results, it is found that the induction system has different induction effects on different genotype of *Brassica napus*. When the karyogene were the same, the induction difference was mainly affected by cytoplasmic genotype. In the same cytoplasmic genotype, the induced differences are mainly affected by the karyogene. Therefore, the induction process of DH inducer line has interaction effects with maternal karyogene and cytoplasmic genotype. At same time, it was found that there was a very significant positive correlation between the genetic similarity rate and homozygous rate of SNP sites of induced F_1_ generation and the maternal parent (Additional file 7), Such as F_1_ of 0068A× Y3560 and the maternal parent genetic similarity of 99.79%, but its SNP sites homozygous rate and parent SNP sites homozygous rate of 98.79, 97.93%, respectively (Additional file 7); F_1_ of 0933A× Y3560 has a genetic similarity of 98.86% with the maternal parent, but its SNP sites homozygous rate was 99.23%, while the maternal parent SNP sites homozygous rate was 98.27% (Additional file 7). It shows that the DH inducer lines can make homozygous parents infinitely closer to homozygous. In addition, there was some heterozygosity in the paternal parent genes of the F_1_ generation, but the heterozygosity varied greatly, ranging from 0.09 to 64.52% (Table 1). For the induced offspring, some individual plants will also have a small amount of paternal gene infiltration (0.09–0.18%). In the induced offspring, all the *pol* CMS and *nap* cytoplasm did not have paternal gene infiltration, while in the induced offspring of *ogu* CMS as the maternal parent, there was low frequency infiltration of the DH inducer gene, and high frequency hybridization. (Fig. 5a, Table 1). These results indicated that the DH inducer line gene was more likely to penetrate into the progeny when the maternal parent containing *ogu* cytoplasm was induced.

### Mechanism analysis of induction of DH inducer line

The study of inducing vivo induced plants to produce haploid or double haploid has been explored in plant such as maize [22–24], *Brassica napus* [17, 18], persian walnut [25]. However, the mechanism of the induction function of the DH inducer lines is not yet clear, and it is generally believed that uniparental chromosome elimination [14, 26, 27] found in maize that inducer genes were infiltrated into haploids Xu et al. [28] confirmed that double fertilization occurred during the induction of maize haploids, and showed that chromosome elimination was the basis of maize haploid inducer; Zhao et al. [24] Found that within 7 days after pollination, most of the chromosomes of the inducible lines were excreted from the cell, and about 44 Mb of paternal parent chromosomal fragments were also found in the haploid offspring, further confirming the phenomenon of chromosome infiltration. Kasha et al. [29] found that hybrids with two sets of parental genomes can be obtained after pollination in barley [30], but the chromosomes of bulbous barley will be selectively lost shortly afterwards [31]. Burk et al. produced haploids in tobacco through distant hybridization [32]. Therefore, based on the investigation results of the mechanism of haploid induction lines, this is similar to our findings in *B. napus* of DH inducer.

In this study, allo-octoploid Y3380 and Y3560 were used for pollination of tetraploid *B. napus*, and the progenies were tetraploid. Except for fertility, the progenies were almost the same as the maternal parents, but were significantly different from the paternal parents. From the results of genotyping (Table 1), the SNP hybridization rate of ZS11 as a paternal parent hybrid is over 96.48%, and the maternal parent infiltration rate is 0.19% ~ 0.27%. And the use of inducer as paternal parent, SNP hybrid highest rate of 64.52%, the lowest was 0%, and the maternal parent infiltration rate is 35.39% ~ 100%, indicating that even if there is hybridization between the inducer and maternal parent, it is not general cross, but partial chromosome or genes cross between the inducer and maternal parent. Therefore, we speculate that the reason for the induction of double haploid in *Brassica napus* may also be the selective elimination of inducer chromosomes. A little part of Chromosome or large fragment cross with the maternal parent may be caused by incomplete or partial chromosome loss of inducer. The infiltration of a small number of inducer genes in the progeny may be caused by gene exchange. Further studies are needed to determine whether the induction mechanism of Y3560 and Y3380 is related to some specific genes.

### Application of DH inducer in *Brassica napus* breeding

Based on the above research results, due to the hybridization or gene infiltration of DH inducer to maternal chromosome (but the genotype is highly consistent with maternal karyogene, the maternal infiltration rate is 90.44 to 99.91%), these conditions may slightly change the maternal inheritance characteristics, and the overall consistency, especially for the same karyogene and different cytoplasmic maternal parent, hybridization and gene infiltration are quite different. We boldly predict that the DH inducer can provide a new model for the innovation of *B. napus* germplasm resources. The innovation the germplasm resources were from DH inducer specific gene infiltration, and we observed in the field that the purple leaf mutation was found during the induction of *ogu* cytoplasmic *B. napus* (the parent does not have purple traits) (Fig. 2c). It is speculated that the infiltration of the inducer gene fragment or transposon during the induction process leads to the acquisition of certain functions of the maternal parent. Therefore, the phenomenon that the infiltration of the inducer gene enables the plant to obtain certain functions can be applied to development of rape germplasm resources, to find in-depth special sites, and to modify the maternal genes at specific sites. The application of rape DH inducer can accelerate and change the rape breeding model, and create new ideas for development of germplasm resources, which have huge application potential and practical value.

## Conclusions

This study explored the induction characteristics of two double-haploid inducible lines of *B. napus*. It was found that when the induced lines were used as the paternal parent to pollinate the maternal parent, the hybrid offspring showed heterogeneous hybridization of the paternal parent gene; and some plants of the induced offspring had a small amount paternal gene infiltration. And according to the different types of maternal parent, there are differences in induction. When the karyogene are the same, the induction differences are mainly affected by cytoplasmic genes; when the cytoplasmic genes are the same, the induction differences are mainly affected by karyogene genes. The induction process of the inducible line interacts with the maternal genotype and cytoplasmic type. Use this interaction effect to provide new insights for the innovation of *B. napus* germplasm resources.

## Methods

### Materials

The DH inducer lines, Y3380 and Y3560 (AAAACCCC, 2n = 8x ≈ 76) are new rapeseed varieties that were artificially bred by of Chengdu Academy of Agriculture and Forestry Sciences in 2011 [18]. These were identified as allo-octoploid by cytology and flow cytometry analysis and were used as the paternal parent. While, maintainer lines 0933B and D717B (*nap* cytoplasm genotype) were used as maternal parent. These maternal lines were crossed with tetraploid generation inbred lines for more than 15 generations. The *pol* CMS lines (0068A, 0933A, D717A) and *ogu* CMS (L0068A, L0933A) having tetraploid number of chromosomes (AACC, 2n = 38) were backcrossed to maintainer lines for 12–15 generations. All types of maternal parents (maintainer lines, *pol* CMS and *ogu* CMS) used in this experiment have the same karyogene, and were different for cytoplasmic genotype (Additional file 8). The collection and breeding of the *B. napus* materials were obtained and used with local permission of China Germplasm Regulation Authorities. The *B.napus* used in this study was commonly grown as oil crop and considered as native species of China and does not fall under the Nagoya protocol. All plant materials including ZS11 (control), sterile, maintainer and induction lines were grown under the same conditions at experimental field in Wenjiang (E103.83, N30.70), Chengdu, China, in October 2016. These materials were artificially pollinated at the flowering stage in March, 2017 and harvested by mixed threshing. All experimental material and their hybrids were grown as triplicate in order to avoid statistical error. In October 2018, all planting material was initially raised in the nursery in pots and later on, were transplanted to the field.

### Flow cytometry

Young leaves of 15 plant of each specific plant materials and their hybrid were we randomly chosen and used for flow cytometric analysis by a previously published protocol [33]. ZS11 was crossed as paternal parent withF_1_ generation (maternal parent) and three randomly selected strains were tested.

### Plant type and fertility identification of induced progeny

According to the characteristics of sterile lines, fertility was used as a marker character for identification for artificial observation in the field. Among them, fertile plants had full anthers and normal pollen, while sterile lines showed different characteristics according to different types of plant. The morphology of *pol* CMS plants was not significantly different from that of fertile plants, but anthers were atrophied, without pollens or contained few pollens at low temperature [34]. The stamens of *ogu* CMS were differentiated with thin anthers that did not produce normal pollen. We identified plant type and counted the number of fertile plants of each F_1_ generation and photographed.

### DNA extraction and PCR amplification

The total genomic DNA of leaves were extracted using the CTAB method. We grinded fresh leaves quickly in liquid nitrogen, added in 900 μl CTAB extract, and then heated in a water bath for 45 min, followed by placing on a shaker (80 rpm) for 15 min for mixing well. Then, centrifuged the samples at 12000 rpm for 10 min, aspirated the supernatant, added an equal volume of pre-cooled isopropanol and placed on ice for 30 min. All samples were then centrifuged at 12000 rpm for 5 min, washed twice with 70% and absolute ethanol, dissolved in 100 uL ddH2O, and finally, DNA quality was detected by 0.8% agarose gel electrophoresis. The PCR reaction program is 94 °C for 4 min, 94 °C for the 30s, TM30s, 72 °C for 45 s, 72 °C for 8 min, 35 Cycle. And 2 to 2.5% agarose gel electrophoresis for detection.

### SNP chip analysis

We used the 6 K Infinium SNP chip, which was evenly distributed across the rapeseed genome (Additional file 15), to identify and analyze the parental gene loci in the progeny. The genome of was *B.napus*_v4.1 (http://brassicadb.org/brad/datasets/pub/Genomes/Brassica_napus/) was used as reference. The quality control of SNP sites were performed with DQC ≥ 0.82 and CR ≥ 95. Finally, 3816 SNP sites with useful polymorphism were screened out, accounting for 75.44% of the total labeled sites. These loci were analyzed by using related software, and the homozygosity rate of individual gene sites and the genetic similarity rate of maternal parents were compared. Genetic distance of maternal parents was calculated using Power Maker V3.25 software. Genetic similarity rate was calculated using a formula e.g., S = Nxy / Nx + Ny (Nxy is the same genotype in the two samples, Nx and Ny are the number of genotypes in X and Y samples, respectively). SNP gene analysis was based on the SNP locus of each pollinated combination that was different but homozygous for the parents. The SNP of the same locus in the offspring were determined using R studio for genotyping (Additional file 11). If the SNP banding pattern in offspring is the same as that of paternal parent, it is judged to be infiltrated and consistent with the maternal parent. While, the SNP was judged to be induced, if a hybrid band type was found in progeny than parental genotype. The 6 K chip detection was done by China Golden Marker Biology Co., Ltd. (Additional file 6).

## Supplementary Information

**Additional file 1.** Flow cytometry diagram of induced line Y3560, maternal parent 0068A and induced offspring.

**Additional file 2.** Flow cytometry results statistics. The ploidy of rape was calculated by fluorescence intensity, and the peak value range represents the fluorescence signal value of mitotic G1 phase of flow cytometry. There are some differences in the fluorescence signal value of each material under the same ploidy, and there significant differences in the fluorescence signal value between ploidy.

**Additional file 3.** Fertility identification results.

**Additional file 4.** Amplification results of SSR primers of some parents and F_1_ individual plants. a: Amplification of SSR specific primer H57 in the paternal inducible line Y3560 (as paternal parent), 0068A (as maternal parent) and F_1_ progeny, 0068A × Y3560 progeny, from left to right are M1–1, M1–2, M1–3, M1–8, M1–9, M1–10; amplification of specific SSR primer H172 in parents and hybrid progeny (0068A × ZS11). b: Amplification of SSR specific primer H57 in the paternal inducible line Y3380 (as paternal parent), L0068A (as maternal parent) and F_1_ progeny, L0068A × Y3380 progeny, from left to right are M4–3, M4–4, M4–5, M4–9; amplification of specific SSR primer H172 in parents and hybrid progeny (L0068A × ZS11). c: Amplification of SSR specific primer H57 in the paternal inducible line (as paternal parent), D717A (as maternal parent) and F_1_ progeny, D717A × Y3560 progeny, from left to right are M39–2, M39–4, M39–6, M39–8, M39–13; D717A × Y3380 progeny, from left to right are M38–1、M38–2、M38–5, M38–9. d: Amplification of specific SSR primer H38 in parents and hybrid progeny (D717A × ZS11). The samples derive from the same experiment and the full-length original gel is included in Additional file 5.

**Additional file 5.** Original image of agarose gel electrophoresis detection. a: Original agarose gel electrophoresis detection image of Fig. 3a-b. b: Original agarose gel electrophoresis detection image of Fig. 3c-d, except that the first band is a duplicate of the maternal parent L0068A, the others are the same as in Fig. 3c-d. c-d: Original agarose gel electrophoresis detection image of Additional file 4.

**Additional file 6.** 6 K SNP chip raw data.

**Additional file 7.** SNP homozygosity rate and inheritance rate.

**Additional file 8. **Test material information. The same number of materials used represents the same karyogene, “A” represents sterile line, “B” represents maintainer line, pol CMS was bred by backcross with 15 generations of maintainer line, and ogu CMS was bred by backcross with 12 generations of the same maintainer line, only cytoplasmic genotype differences, but 0068 and 0933 have similar sources. The collection and breeding of the *Brassica napus* materials we use has obtained local permits in China, which is in line with national policies. The *Brassica napus* used in this study is commonly grown oil crop used species native to China and does not fall under the Nagoya protocol.

**Additional file 9.** Genotyping diagram of induced line before and after induction of 0068. a-c: respectively, are the genotyping diagrams of parent and progeny before and after induction of 0068A × Y3560, L0068A × Y3380, 0068A × ZS11, and the numbered band M is the progeny plant.

**Additional file 10.** Genotyping diagram of induced line before and after induction of D717. a-e: for the genotyping diagrams of parents and progeny before and after induction of D717A × Y3560, D717A × Y3380, D717B × Y3560, D717A × Y3380, and D717A × ZS11, respectively. The band M in the number is the progeny plant.

**Additional file 11.** Genotyping raw data.

**Additional file 12.** Statistics of the number of SNPs of heterozygous loci in the hybrid F_1_ generation.

**Additional file 13.** F_1_ generation paternal parent infiltrated SNP site.

**Additional file 14.** Statistics of identification results of different identification methods.

**Additional file 15. **SNP site distribution map. The blue dots represent the distribution of SNP sites on chromosomes A01-A10 and C01-C09 in *Brassica napus*. The 6 k (total 5127 sites) SNP chip is well distributed on 19 chromosomes.

## Data Availability

The datasets supporting the conclusions of this article are included within the article and its additional files.
