# Supplementary material for: Maternal karyogene and cytoplasmic genotype affect the induction efficiency of doubled haploid inducer in Brassica napus
Source: BMC Plant Biol. 2021 May 3;21:207. doi: 10.1186/s12870-021-02981-z (PMC8091669; doi:10.1186/s12870-021-02981-z)

Additional file 1. Flow cytometry diagram of induced line Y3560, maternal parent 0068A and induced offspring.

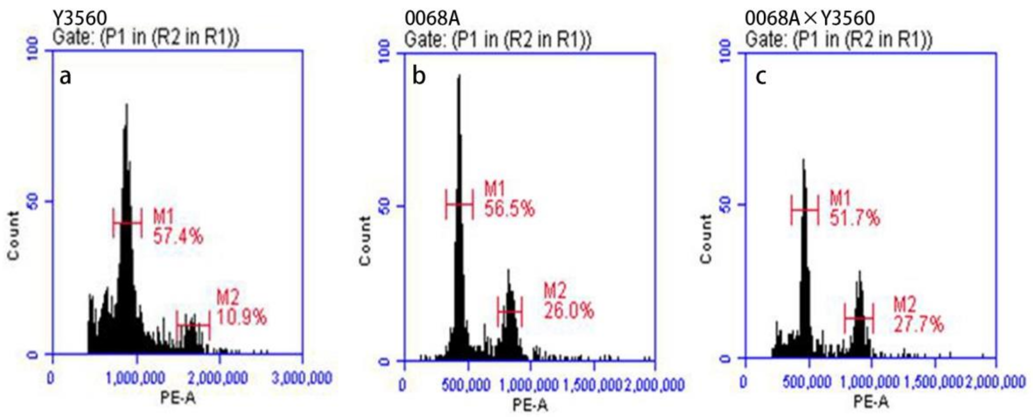

Supplement: Supplementary file 1 — Additional file 1. Flow cytometry diagram of induced line Y3560, maternal parent 0068A and induced offspring. [file 12870_2021_2981_MOESM1_ESM.pdf]
