# Supplementary material for: Maternal karyogene and cytoplasmic genotype affect the induction efficiency of doubled haploid inducer in Brassica napus
Source: BMC Plant Biol. 2021 May 3;21:207. doi: 10.1186/s12870-021-02981-z (PMC8091669; doi:10.1186/s12870-021-02981-z)
Supplement: Supplementary file 4 — Additional file 4. Amplification results of SSR primers of some parents and F1 individual plants. a: Amplification of SSR specific primer H57 in the paternal inducible line Y3560 (as paternal parent), 0068A (as maternal parent) and F1 progeny, 0068A × Y3560 progeny, from left to right are M1–1, M1–2, M1–3, M1–8, M1–9, M1–10; amplification of specific SSR primer H172 in parents and hybrid progeny (0068A × ZS11). b: Amplification of SSR specific primer H57 in the paternal inducible line Y3380 (as paternal parent), L0068A (as maternal parent) and F1 progeny, L0068A × Y3380 progeny, from left to right are M4–3, M4–4, M4–5, M4–9; amplification of specific SSR primer H172 in parents and hybrid progeny (L0068A × ZS11). c: Amplification of SSR specific primer H57 in the paternal inducible line (as paternal parent), D717A (as maternal parent) and F1 progeny, D717A × Y3560 progeny, from left to right are M39–2, M39–4, M39–6, M39–8, M39–13; D717A × Y3380 progeny, from left to right are M38–1、M38–2、M38–5, M38–9. d: Amplification of specific SSR primer H38 in parents and hybrid progeny (D717A × ZS11). The samples derive from the same experiment and the full-length original gel is included in Additional file 5. [file 12870_2021_2981_MOESM4_ESM.pdf]

Additional file 4 Amplification results of SSR primers of some parents and F1 individual plants.

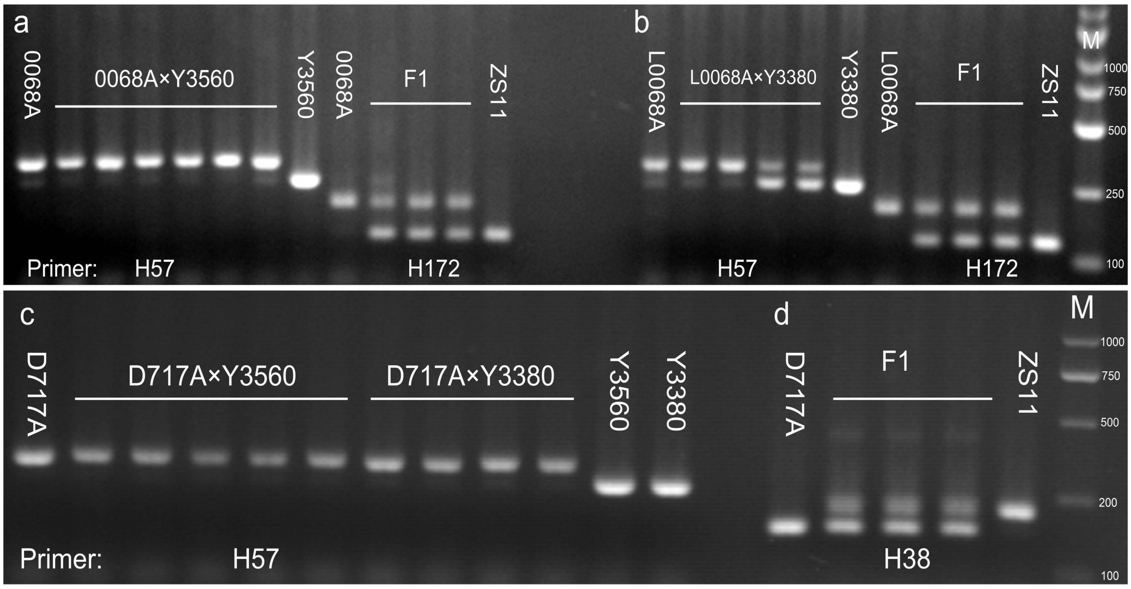

a: Amplification of SSR specific primer H57 in the paternal inducible line Y3560 (as paternal parent), 0068A (as maternal parent) and F1 progeny, 0068A × Y3560 progeny, from left to right are M1-1, M1-2, M1-3, M1-8, M1-9, M1-10; amplification of specific SSR primer H172 in parents and hybrid progeny (0068A×ZS11). **b**: Amplification of SSR specific primer H57 in the paternal inducible line Y3380 (as paternal parent), L0068A (as maternal parent) and F1 progeny, L0068A × Y3380 progeny, from left to right are M4-3, M4-4, M4-5, M4-9; amplification of specific SSR primer H172 in parents and hybrid progeny (L0068A×ZS11). **c**: Amplification of SSR specific primer H57 in the paternal inducible line (as paternal parent), D717A (as maternal parent) and F1 progeny, D717A × Y3560 progeny, from left to right are M39-2, M39-4, M39-6, M39-8, M39-13; D717A × Y3380 progeny, from left to right are M38-1、M38-2、M38-5, M38-9. **d**: Amplification of specific SSR primer H38 in parents and hybrid progeny (D717A×ZS11). The samples derive from the same experiment and the

full-length original gel is included in Additional file 5.
