# Supplementary material for: Maternal karyogene and cytoplasmic genotype affect the induction efficiency of doubled haploid inducer in Brassica napus
Source: BMC Plant Biol. 2021 May 3;21:207. doi: 10.1186/s12870-021-02981-z (PMC8091669; doi:10.1186/s12870-021-02981-z)
Supplement: Supplementary file 5 — Additional file 5. Original image of agarose gel electrophoresis detection. a: Original agarose gel electrophoresis detection image of Fig. 3a-b. b: Original agarose gel electrophoresis detection image of Fig. 3c-d, except that the first band is a duplicate of the maternal parent L0068A, the others are the same as in Fig. 3c-d. c-d: Original agarose gel electrophoresis detection image of Additional file 4. [file 12870_2021_2981_MOESM5_ESM.pdf]

Additional file 5 Original image of agarose gel electrophoresis detection

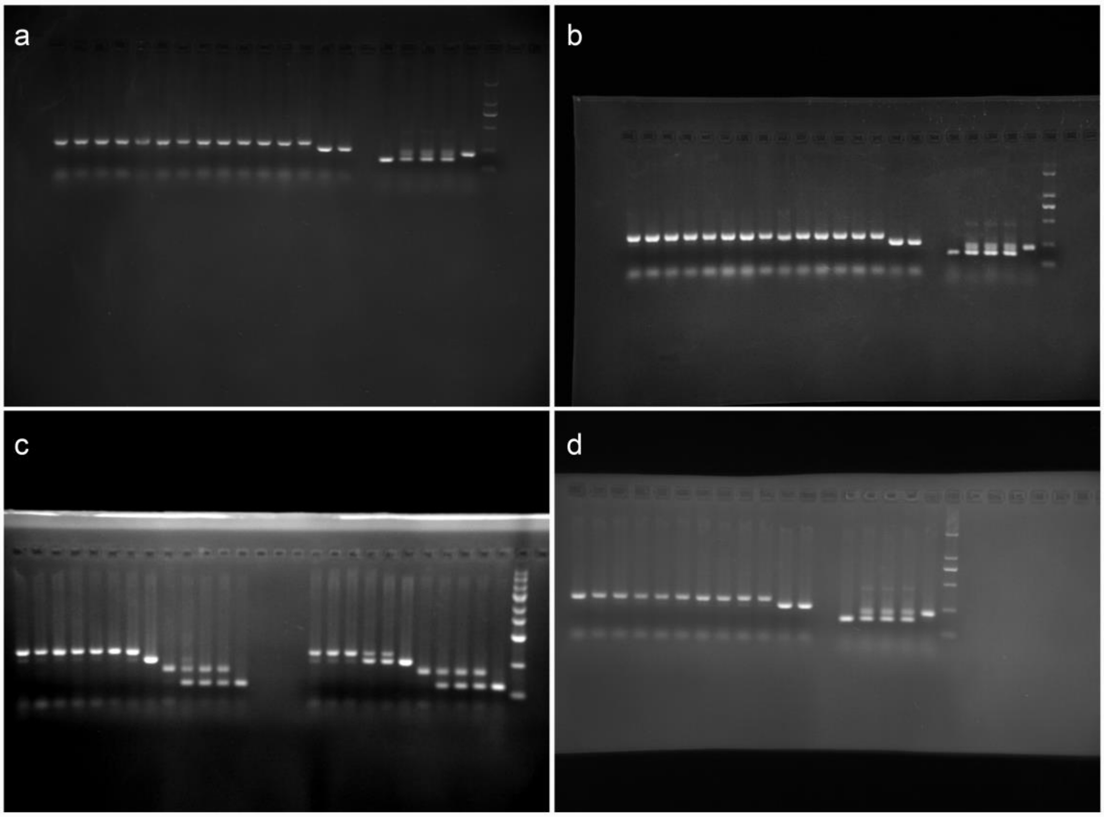

a: Original agarose gel electrophoresis detection image of Fig3a-b. **b**: Original agarose gel electrophoresis detection image of Fig3c-d, except that the first band is a duplicate of the maternal parent L0068A, the others are the same as in Fig 3c-d. **c-d**: Original agarose gel electrophoresis detection image of Additional file 4.
