# Supplementary material for: Maternal karyogene and cytoplasmic genotype affect the induction efficiency of doubled haploid inducer in Brassica napus
Source: BMC Plant Biol. 2021 May 3;21:207. doi: 10.1186/s12870-021-02981-z (PMC8091669; doi:10.1186/s12870-021-02981-z)
Supplement: Supplementary file 9 — Additional file 9. Genotyping diagram of induced line before and after induction of 0068. a-c: respectively, are the genotyping diagrams of parent and progeny before and after induction of 0068A × Y3560, L0068A × Y3380, 0068A × ZS11, and the numbered band M is the progeny plant. [file 12870_2021_2981_MOESM9_ESM.pdf]

Additional file 9. Genotyping diagram of induced line before and after induction of 0068.

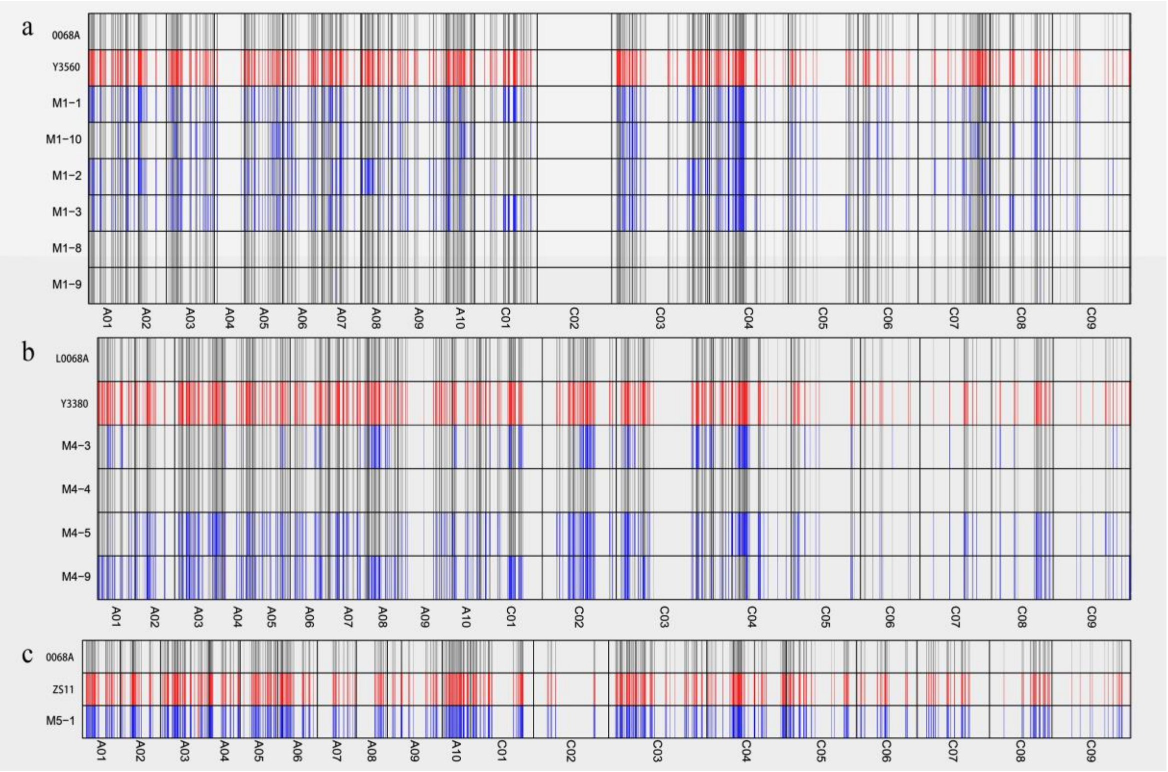

a-c: respectively, are the genotyping diagrams of parent and progeny before and after induction of 0068A ×Y3560, L0068A ×Y3380, 0068A ×ZS11, and the numbered band M is the progeny plant.
