# Supplementary material for: Maternal karyogene and cytoplasmic genotype affect the induction efficiency of doubled haploid inducer in Brassica napus
Source: BMC Plant Biol. 2021 May 3;21:207. doi: 10.1186/s12870-021-02981-z (PMC8091669; doi:10.1186/s12870-021-02981-z)
Supplement: Supplementary file 10 — Additional file 10. Genotyping diagram of induced line before and after induction of D717. a-e: for the genotyping diagrams of parents and progeny before and after induction of D717A × Y3560, D717A × Y3380, D717B × Y3560, D717A × Y3380, and D717A × ZS11, respectively. The band M in the number is the progeny plant. [file 12870_2021_2981_MOESM10_ESM.pdf]

Additional file 10. Genotyping diagram of induced line before and after induction of D717.

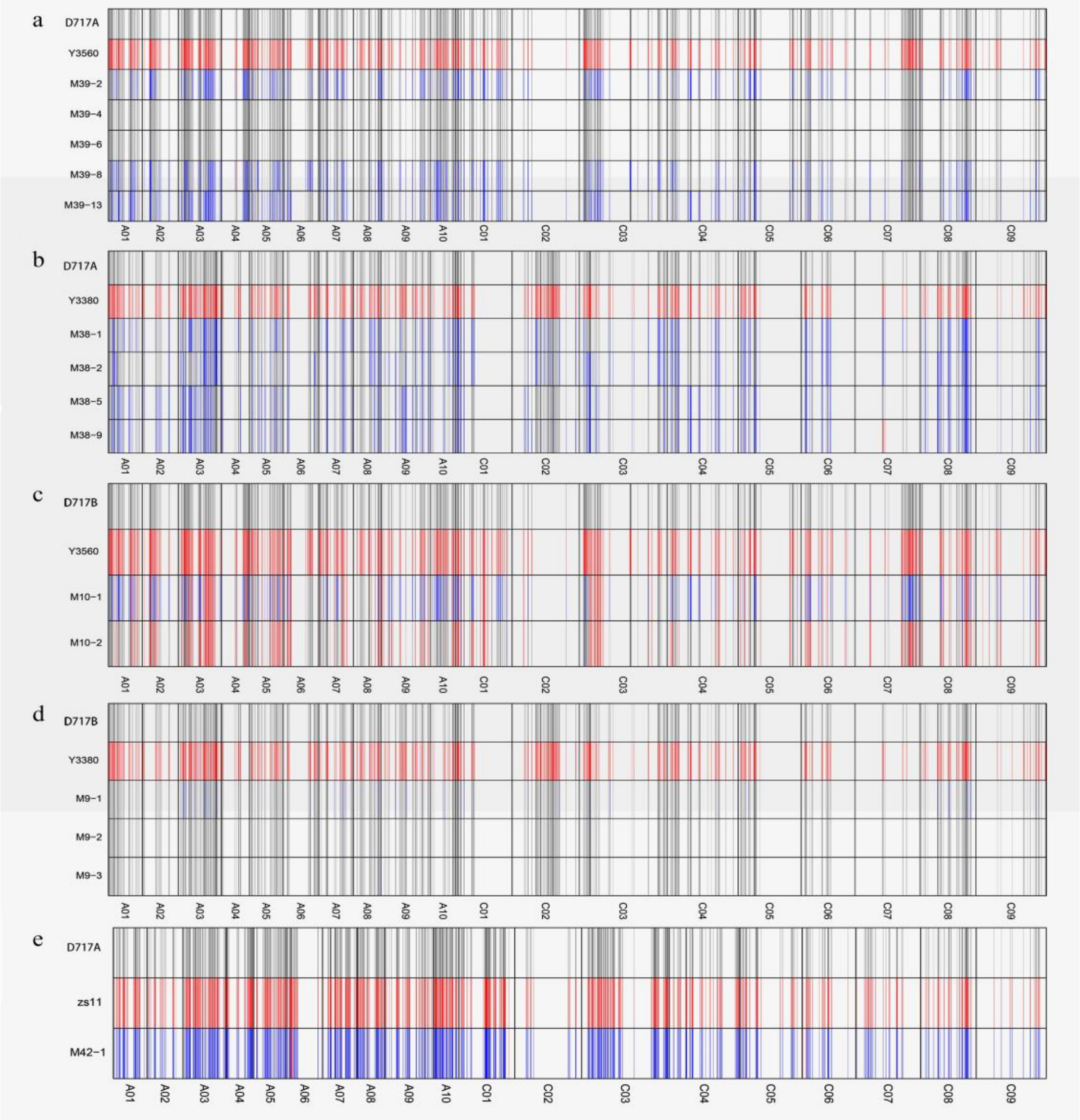

a-e: for the genotyping diagrams of parents and progeny before and after induction of D717A×Y3560, D717A×Y3380, D717B×Y3560, D717B×Y3380, and D717A×ZS11, respectively. The band M in the number is the progeny plant.
