# Supplementary material for: Maternal karyogene and cytoplasmic genotype affect the induction efficiency of doubled haploid inducer in Brassica napus
Source: BMC Plant Biol. 2021 May 3;21:207. doi: 10.1186/s12870-021-02981-z (PMC8091669; doi:10.1186/s12870-021-02981-z)
Supplement: Supplementary file 15 — Additional file 15. SNP site distribution map. The blue dots represent the distribution of SNP sites on chromosomes A01-A10 and C01-C09 in Brassica napus. The 6 k (total 5127 sites) SNP chip is well distributed on 19 chromosomes. [file 12870_2021_2981_MOESM15_ESM.pdf]

**Additional file 15.** SNP site distribution map.

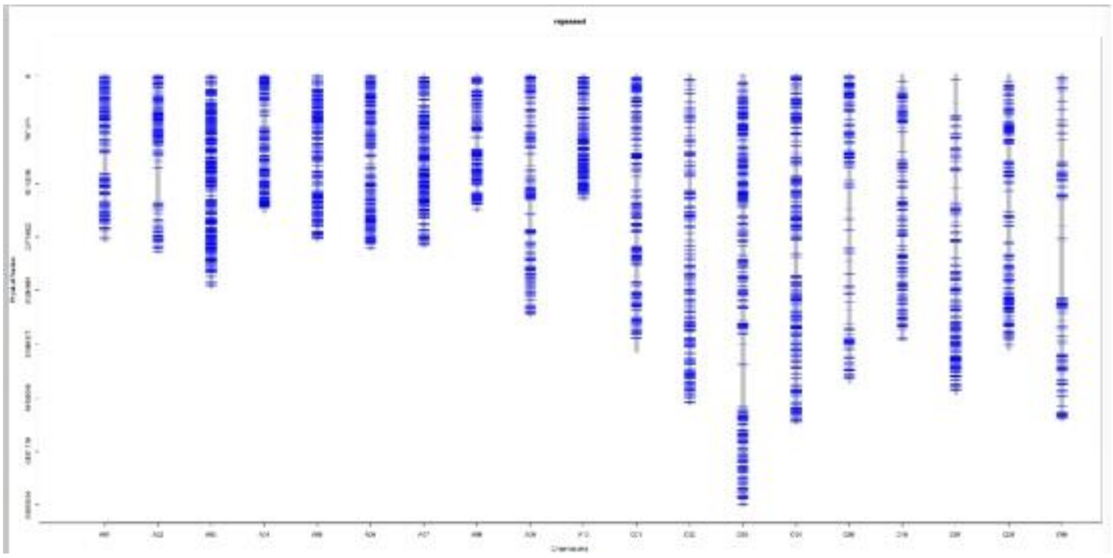

The blue dots represent the distribution of SNP sites on chromosomes A01-A10 and C01-C09 in *Brassica napus*. The 6k (total 5127 sites) SNP chip is well distributed on 19 chromosomes.
